# Substance use patterns in an adolescent psychiatric unit in Johannesburg, South Africa

**DOI:** 10.4102/sajpsychiatry.v30i0.2198

**Published:** 2024-01-31

**Authors:** Vuyani W. Nxumalo, Yvette M. Nel

**Affiliations:** 1 Department of Psychiatry, Faculty of Health Sciences, University of the Witwatersrand, Johannesburg, South Africa

**Keywords:** adolescent, mental health, inpatient, substance use, prevalence

## Abstract

**Background:**

Substance use among adolescents carries a significant public health and socioeconomic burden with potential long-term consequences for the adolescent substance user (SU). Adolescents with mental health challenges are vulnerable to substance use and substance use worsens outcomes in this population.

**Aim:**

This study aimed to describe the substance use patterns among inpatients admitted to a specialised tertiary adolescent inpatient unit in Johannesburg over a 4-year period.

**Setting:**

This study was conducted at the Tara H. Moross Centre (Tara Hospital), in Johannesburg in the Gauteng province of South Africa.

**Methods:**

This was a retrospective comparative record review of all patients admitted to the adolescent unit over the 4 years.

**Results:**

A lifetime history of substance use was documented in 44.1% (*n* = 52) of the 118 patient’s records included in the final analysis. Cannabis was the most frequently used substance (*n* = 36, 69.2%). There were significant differences between the SU and substance nonuser groups regarding family structure (*p* = 0.012), family history of substance abuse (*p* = 0.046) and conflict within the family (*p* < 0.001).

**Conclusion:**

The high prevalence of substance use in this sample demonstrates the dual burden of mental health disorders and substance use in an adolescent treatment programme in Johannesburg. Primary caregiver burden and relational difficulties within the family unit should be observed for planned multidisciplinary interventions.

**Contribution:**

The findings of this review provide an update on the pattern and prevalence of substance use among this adolescent mental healthcare user group, highlighting potential therapeutic targets.

## Introduction

Substance use has several multisectoral societal consequences, with a disproportionately higher prevalence of substance use in youth and men globally.^1^ The annual prevalence of substance use worldwide for persons between the ages of 15 years and 64 years was estimated to be 5.6% in 2020,^1^ with 13.6% of these substance users (SU) reported to be suffering from a substance-related disorder.^1^ The European School Survey Project on Alcohol and Other Drugs 2019 report noticed a lifetime prevalence of illicit drug use among adolescents of age 15 years – 16 years at 17%, with cannabis reported as the most easy to obtain illicit substance.^2^ Cannabis is the most widely used controlled substance worldwide, with use particularly prevalent in Africa.^1^ There is a higher prevalence of cannabis use in youth populations compared with the entire population globally.^1^ A recent systematic review found alcohol, cannabis and tobacco to be the most common drugs of use among adolescents.^3^

Early onset substance use has substantial implications for the development of adult substance use disorders, drug-related deaths and drug-related treatment, as well as for other important psychosocial consequences.^1^ Adult substance use disorders often have their origins in adolescence.^4^ In addition, adolescent onset of substance use increases the likelihood of developing a substance use disorder.^5^ Adolescent substance use has also been shown to increase the risk of developing other psychiatric disorders.^6^ There is a complex and often bidirectional relationship between substance use and mental illness.^7,8^ Substances may be used to self-medicate psychiatric symptoms, notably anxiety and depression, and substance use may also exacerbate or induce a variety of mental health conditions.^8^ There is evidence to suggest that adolescent substance use is associated with poor school performance, subsequent school failure and resulting higher levels of unemployment in adulthood. This may lead to increased involvement in illegal activities.^9^

Theoretical underpinnings describing the factors influencing the increased risk of substance use, which has been observed in the adolescent period include a propensity towards novelty seeking behaviours and activation of reward system neural pathways.^10^ Development and expansion of neuronal dopaminergic connectivity in the adolescent brain, particularly involving the prefrontal and limbic regions, with an overall increase in white matter connectivity have been described in the developing adolescent brain.^10^ Adolescents have been found to have an immature prefrontal cortex, resulting in poor impulse control with a tendency towards seeking out new experiences, including substance experimentation.^6,10^ Additionally, psychosocial and clinical factors have been identified, which increase the risk of adolescent substance use.^11,12^ Familial, social and individual risk factors contributing to adolescent substance use have been highlighted previously.^11^ Familial factors included child abuse and neglect, a positive history within the family of substance abuse, parent–child relational difficulties and divorce of parents. Social factors have been described as bullying, gangsterism and affiliation with other adolescents with a history of defiant behaviour. Individual factors relate to a diagnosis of a clinical mental health condition, including attention deficit hyperactivity disorder (ADHD) and depression.^11^ There is a threefold higher risk of developing a substance use disorder in those with comorbid ADHD compared with those with another mental disorder.^11^ This increased risk decreases with successful treatment of ADHD.^11^

The prevalence of substance use has been reported to be higher in adolescents with mental illness when compared with the general adolescent population.^13,14,15^ Data suggest that cannabis and alcohol are the most prevalent substances used by adolescents in South Africa in general, as well as in psychiatric adolescent populations.^15,16,17,18^ A recent international study similarly reported cannabis as the most prevalent illicit substance used among adolescent psychiatric inpatients.^19^ In 2008, Hollen et al. noticed substance use comorbidities in 25% of adolescent inpatient mental healthcare users.^13^ Niethammer and Frank likewise reported high rates of substance use in Germany, with 44% of adolescent mental healthcare users in an inpatient unit reporting regular alcohol use and 40% reporting illegal substance use.^20^ A recent Polish study found a lifetime use prevalence of 34% of illicit substances among adolescent hospitalised inpatients.^19^ South African literature has likewise demonstrated high levels of substance use among adolescent mental healthcare users, with lifetime prevalence of cannabis use reported as between 55.6% and 61.4% among inpatient adolescent mental healthcare users admitted in Durban.^14,15^ In the Western Cape, Lachman et al. reported a high lifetime prevalence of substance use among inpatient mental healthcare users with first episode psychosis.^21^

### Aim and objectives

The aim of this study was to describe the pattern of substance use among inpatients admitted to a specialised tertiary adolescent unit in Johannesburg over a 4-year period. The objectives of this study were to describe the prevalence of lifetime reported substance use, to describe the pattern of this substance use and to describe and compare the socioclinical profiles of the patients with and without a substance use history.

## Research methods and design

### Setting

The Tara H. Moross Centre (Tara Hospital) is located in Johannesburg in the Gauteng Province of South Africa. The hospital is a specialised psychiatric hospital with both inpatient and outpatient services directed at adolescents with mental health disorders. There are 12 adolescent inpatient beds.

### Study design and sample population

This study was a retrospective comparative record review conducted at Tara Hospital. All the available files of adolescent patients between the ages of 13 and 18 years who were admitted at the Tara Hospital’s adolescent unit at any time over a 4-year period from 01 January 2012 until 31 December 2015 were included in the study, with the only exclusion being files which were not traceable, and were therefore excluded from analysis.

The study’s main outcome was to determine the prevalence of substance use in adolescent inpatients at Tara Hospital. Information about substance use was obtained from the admission clerking sheet and ward file. Substance use, for this study’s purpose, was defined as lifetime use of any psychoactive substance, as observed in the patient’s history. This was ascertained from any one or more of the following documented in the clinical record: a self-report of substance use or misuse, a diagnosis of substance-related disorder, a positive multidrug urine screen during admission and collateral information. Multidrug urine test screening for all the inpatients was not performed routinely. Information on diagnosis and clinical and demographic data was obtained from the clinical file. No rating scales were used.

### Data analysis

All data were manually collected and entered into an electronic spreadsheet. The *Χ*^2^ test was used to assess the relationships between categorical variables and SU group. Fisher’s exact test was used for 2 × 2 tables or where the requirements for the *Χ*^2^ test could not be met. The relationship between continuous variables and SU group was assessed by the *t*-test. Where the data did not meet the assumptions of the test, a nonparametric alternative, the Wilcoxon rank sum test was used. The relationship between study variables and SU status was assessed by binomial regression. Categories with *n* < 10 were not included in the analysis. Variables that were significant univariately were combined into a multivariable model, after examining each pair of variables for possible confounding using the chi-squared test (or Fisher’s exact test for 2 × 2 tables). A value of Cramer’s V (or the phi coefficient for Fisher’s exact test) > 0.60 was regarded as too strong an association to include both variables in a multivariable model. Nonsignificant variables were sequentially removed from the multivariable model. The 5% significance level was used. Statistical Analysis System (SAS, version 9.4 for Windows) was used for all analysis.

### Ethical considerations

This was a retrospective study and confidentiality was maintained during data collection, analysis and storage of data. The patients’ personal identifying data were not recorded. The original protocol was approved by the University of the Witwatersrand Postgraduate Committee. Ethical clearance was obtained from the University of the Witwatersrand Human Research Ethics Committee (M170410); informed consent was not required by the ethics committee because of the retrospective nature of data collection. Permission to conduct this study was also obtained from the Hospital Research Committee.

## Results

Overall, 140 patients were admitted to the Tara Hospital adolescent ward during the study period, but there were 17 missing files and 5 repeat admissions. The final sample was therefore 118 patients.

The sociodemographic profile of the SU and substance nonuser (SNU) groups is presented in Table 1. There were no significant differences in the gender profiles or ethnic grouping of the SU and SNU groups. There was a significant difference in the age of the SU and SNU groups, with the SU group being slightly older than the SNU group (*p* = 0.014). There were no statistically significant differences between the two groups with respect to the type of schooling (*p* = 0.077).

**TABLE 1:** Sociodemographic profile of substance users and substance nonusers.

Clinical variable	SU (*N* = 52)				SNU (*N* = 66)				*P*
	*n*	%	median	IQR	*n*	%	median	IQR	
**Age (years)**	-	-	16	15–17	-	-	15	14–16	0.01*#
**Gender**									
Male	24	46.2	-	-	31	47.0	-	-	> 0.999
Female	28	53.8	-	-	35	53.0	-	-	-
**Race**									
White people	20	38.5	-	-	30	45.5	-	-	0.691$
African people	24	46.2	-	-	29	43.9	-	-	-
Mixed race people	5	9.6	-	-	3	4.5	-	-	-
Indian people	3	5.8	-	-	4	6.1	-	-	-
**Main caregiver**									
Both parents	15	29.4	-	-	36	54.5	-	-	0.012*
Father	2	3.9	-	-	3	4.5	-	-	-
Mother	24	47.1	-	-	16	24.2	-	-	-
Grandparents	5	9.8	-	-	9	13.6	-	-	-
Adoptive parents	3	5.9	-	-	0	0	-	-	-
Siblings	1	2.0	-	-	0	0	-	-	-
Other	1	2.0	-	-	0	0	-	-	-
Other relatives	1	2.0	-	-	2	3.0	-	-	-
**Years of schooling**	-	-	9	8–10	-	-	8	7–9	0.174
**Type of schooling**									
Mainstream	38	74.5	-	-	54	84.4	-	-	0.077
Special	1	2.0	-	-	5	7.8	-	-	-
Remedial	5	9.8	-	-	2	3.1	-	-	-
Other	1	2.0	-	-	2	3.1	-	-	-
Not in school	7	13.7	-	-	3	4.7	-	-	-
**Psychosocial stressors**									
Academic difficulties	23	44.2	-	-	31	47.0	-	-	0.142
Divorce and/or separation of parents	27	51.9	-	-	21	31.8	-	-	0.038*
Conflict with parents	32	61.5	-	-	17	25.8	-	-	< 0.001*
Poor peer relationships	15	28.8	-	-	14	21.2	-	-	0.391
Parental conflict	10	19.2	-	-	15	22.7	-	-	0.823
Early parental loss	10	19.2	-	-	8	12.1	-	-	0.310
None reported	0	0.0	-	-	2	3.0	-	-	0.500

*Source:* Adapted from original MMED research report by first author VN. Available at https://wiredspace.wits.ac.za/items/f5465b91-36cf-48e6-8a97-c5057c51f15b

All *p*-values calculated using Fisher’s exact test except where otherwise stated.

SU, substance user; SNU, substance nonuser; IQR, interquartile range; mdn, median.

* , significant;

# , *t*-test;

$ , χ^2^ test.

**FIGURE 1:**
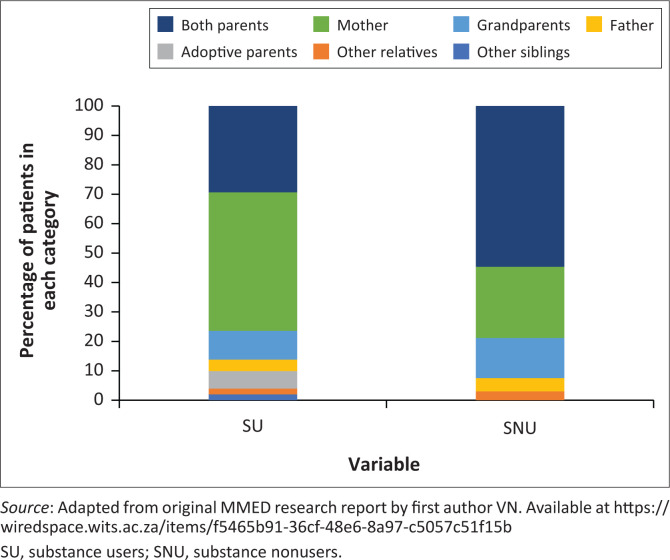
Caregivers in substance users versus substance nonusers.

There was a significant difference between the two groups regarding the main caregiver patterns (*p* = 0.012). Single-parent households were more common in the SU group, with 47.1% (*n* = 24) of SU cared for by the mother alone, compared to 24.2% (*n* = 16) in the SNU group. Furthermore, more SNU had both parents involved in their care, with 54.5% (*n* = 36) cared for by both parents compared to 29.4% (*n* = 15) in the SU group. Substance users were also more likely to have reported conflict with parents as a stressor compared to SNU (Table 2 and Figure 1). More SU came from households where there was ‘divorce or separation of parents’ when compared to SNU (Table 2, Figure 1).

**TABLE 2:** Clinical profile of substance users and substance nonusers.

Clinical variable	SU (*N* = 52)		SNU (*N* = 66)		*P*
	*n*	%	*n*	%	
**Psychiatric diagnosis**					
Mood and anxiety	37	71.2	46	69.7	> 0.999
Psychotic disorder	17	32.7	18	27.3	0.550
Personality disorder	19	36.5	15	22.7	0.111
ADHD	10	19.2	11	16.7	0.812
Disruptive disorder	10	19.2	6	9.1	0.176
Substance use disorder	15	28.8	-	-	-
Learning disorder	3	5.8	8	12.1	0.341
Intellectual disorder	0	0.0	3	4.5	0.252
**Family history of psychiatric condition (*n*= 114)**					
Yes	31	62.0	30	46.9	0.133
No	19	38.0	34	53.1	-
Unknown	2	3.85	2	3.03	-
**Family history of substance use (*n* = 106)**					
Yes	25	53.2	19	32.2	0.046*
No	22	46.8	40	67.8	-
Unknown	5	9.62	7	10.6	-

*Source:* Adapted from original MMED research report by first author VN. Available at https://wiredspace.wits.ac.za/items/f5465b91-36cf-48e6-8a97-c5057c51f15b

All *p*-values calculated using Fisher’s exact test.

ADHD, attention deficit hyperactivity disorder; SU, substance users; SNU, substance nonusers.

* , significant.

There were no significant differences between the SNU and SU groups with regard to clinical diagnostic categories. However, there was a significant association between the SU group and SNU group and the presence of a family history of substance use. The proportion of patients with a family history of substance use was higher in the SU group (*n* = 25, 53.2%) compared to the SNU group (*n* = 19, 32.2%; *p* = 0.046).

All significant variables were included in the further multivariable analysis. There were no confounders observed. Only conflict with parents remained significant in the multivariable model (relative risk [RR] 2.25, 95% CI: 1.48–3.44).

**FIGURE 2:**
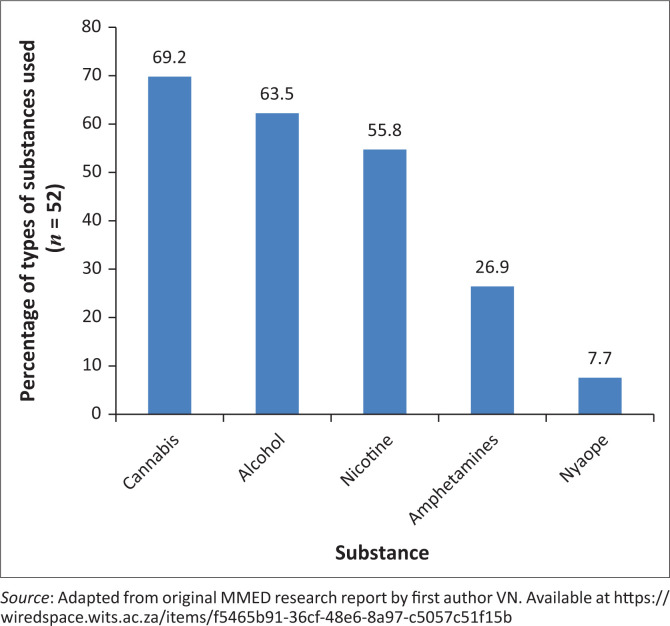
Type of substance used.

The lifetime prevalence of substance use as documented in the clinical record was 44.1% (*n* = 52) with the median age at first use of substances documented as 14 years (IQR [interquartile range] 13 years – 15 years). Of the patients reporting substance use, 46.2% (*n* = 24) were male and 53.8% (*n* = 28) were female. The median duration of substance use was 20 months with minimum duration of 1 month and maximum of 72 months (IQR 11–24 months). Most patients were introduced to substances by their peer group (*n* = 38, 86.4%). Cannabis was the most commonly used substance (*n* = 36, 69.2%), followed by alcohol (*n* = 33, 63.5%), nicotine (*n* = 29, 55.8%), amphetamines (including methamphetamine) (*n* = 14, 26.9%) and ‘nyaope’ (*n* = 4, 7.7%) (a mixture of cannabis and heroine and other substances, also known as ‘sugars’ or ‘*whoonga*’) (Figure 2). Substance use was not limited to one type of substance, and while 33% used only one substance, 67% used two or more substances.

## Discussion

This retrospective review describes the pattern of substance use in an adolescent inpatient population at Tara Hospital over a 4-year period. Almost half of the adolescents admitted to this psychiatric unit had used substances. The prevalence of substance use in this sample group of 44.1% was slightly higher than previously described in a similar South African report, published in 2017.^18^ There has been a range of prevalence rates reported in the literature, influenced by definitions of substance use and substance use disorders. The findings of this report are however broadly in keeping with other local and international studies, reflecting an elevated level of substance use in adolescents with psychiatric disorders and therefore a high burden of comobidity.^13,14,15,20,21^

Cannabis and alcohol were found to be the most used substances at 69.2% and 63.5%, respectively. This is in line with previous local research on adolescent mental healthcare users, with cannabis and alcohol as being the most common drugs of abuse in several centres within South Africa. Specifically, Taukoor et al. found alcohol to be the most frequently used substance, and after that, cannabis, in adolescents with mental illness in Durban,^18^ while Paruk et al.^15^ in Durban and Lachman et al.^21^ in Cape Town observed cannabis to be the most commonly used substance among adolescent mental healthcare users. The South African Community Epidemiology Network on Drug Use (SACENDU) general population report of 2016 found an increase in cannabis use in Gauteng province, making cannabis and then alcohol the most common drugs of abuse.^22^ This finding is also in line with international literature on the patterns of substance use in the general population, with cannabis reported to be the most common illicit drug of abuse in Africa, as well as internationally.^1^

Although this study primarily measured lifetime substance use and not substance use disorder, a diagnosis of substance use disorder was recorded in some of the clinical records (12.7%). Rates of substance use are expected to be higher than a diagnosis of substance use disorder. A comparison of the prevalence of a substance use disorder diagnosis with the rates of substance use disorder diagnosis reported in other similar studies (25% – 54%) suggests that substance use disorder may have been underdiagnosed in this sample particularly considering the 44.1% prevalence of lifetime substance use.^13,21^

There were several significant sociodemographic related factors associated with substance use in this sample in keeping with what is known about adolescent substance use. These factors correlate with findings from previous research carried out on adolescent populations with mental health disorders, which found elevated levels of conflict within families with adolescent substance use.^14,21,23,24,25,26^ The family characteristics differed significantly between the SU and SNU groups, particularly with regard to primary caregiver patterns. While only 29.4% of SU groups were cared for by both parents, there was also a higher prevalence of single mothers in the SU group (47.1%) in contrast to the SNU group. Previous research has demonstrated an association between increased adolescent substance use and being raised in single-parent households.^25,27^

There were no associations observed between diagnosis, as grouped for the purpose of data capture and analysis, and substance use or nonuse. The ‘mood and anxiety disorders’ category was the most common diagnostic grouping for both the SU and nonuser groups. The grouping of conditions in the study might have influenced any potential associations of diagnosis and substance use. Of note, Lachman et al. previously found higher rates of substance-induced psychosis than schizophrenia; however, in the current sample, substance-induced disorders were grouped with the relevant mood, anxiety or psychotic disorder categories. Although conduct disorder has been previously strongly associated with substance use, this was not found in the current sample.^13^ This may also have been influenced by the grouping of conduct disorder with other ‘disruptive, impulse control and conduct disorders’.

Substance use in adolescents has been repeatedly associated with a family history of substance use, particularly adolescent exposure to parental substance use.^21,26,28^ Similarly, there was a significant association between a lifetime history of substance use and a family history of substance use in the current sample. Ali et al. observed specifically that maternal substance use was significantly associated with adolescent substance use.^28^ It has been theorised that increased parent–child relational difficulties in households where there is parental substance use promote adolescent substance use, as does parental acceptance of adolescent substance use.^29^

### Limitations

This study used one research site, Tara Hospital, in Johannesburg and is therefore not generalisable to a broader South African context. Retrospective data were used. There was no routine use of multidrug urine tests in the files, so most of the information was based on self-report and collateral, which is subject to both recall and social desirability bias. No rating scales were used in the clinical files. The broad categorical grouping of clinical diagnosis for data collection and analysis limited the depth of analysis regarding individual diagnosis and substance use. The once-off use of substance, or experimentation, which can be regarded as a normal part of adolescent behaviour was not further explored in this study. The data collected for this study cover the 4-year period of 2012–2015 and as such do not describe the current pattern of substance use in this setting.

## Conclusion

The use of substances in adolescents has the potential to induce, complicate and exacerbate mental health conditions. The high prevalence of lifetime substance use in this population demonstrates the need for targeted interventions, noting family and socioenvironmental stressors, to address substance use among adolescent mental healthcare users. Therapeutic targets should address conflict within the family unit, as well as provide supportive interventions for single-parent households. In addition, given the findings in this sample which add to the existing literature supporting the association of a family history of substance use with adolescent substance use, mentoring parents who are SU in their parenting role, to reduce generational transmission of substance use, may be another therapeutic target. Dual diagnosis should be observed and addressed in the planning and implementation of treatment programmes, which are designed for adolescent mental healthcare users.
